# Metabolite profiling identifies a signature of tumorigenicity in hepatocellular carcinoma

**DOI:** 10.18632/oncotarget.25525

**Published:** 2018-06-01

**Authors:** Shamir Cassim, Valérie-Ann Raymond, Benoit Lacoste, Pascal Lapierre, Marc Bilodeau

**Affiliations:** ^1^ Laboratoire d’hépatologie cellulaire, Centre de recherche du Centre hospitalier de l'Université de Montréal (CRCHUM), Montréal, QC, Canada; ^2^ Département de médecine, Université de Montréal, Montréal, QC, Canada

**Keywords:** liver, hepatocellular carcinoma, glucose, tumorigenicity, metabolic signature

## Abstract

HCC (Hepatocellular carcinoma) cells exhibit greater metabolic plasticity than normal hepatocytes since they must survive in a dynamic microenvironment where nutrients and oxygen are often scarce. Using a metabolomic approach combined with functional *in vitro* and *in vivo* assays, we aimed to identify an HCC metabolic signature associated with increased tumorigenicity and patient mortality. Metabolite profiling of HCC Dt81Hepa1-6 cells revealed that their increased tumorigenicity was associated with elevated levels of glycolytic metabolites. Tumorigenic Dt81Hepa1-6 also had an increased ability to uptake glucose leading to a higher glycolytic flux that stemmed from an increased expression of glucose transporter GLUT-1. Dt81Hepa1-6-derived tumors displayed increased mRNA expressions of glycolytic genes, *Hypoxia-inducible factor-1alpha* and of *Cyclin D1*. HCC tumors also displayed increased energy charge, reduced antioxidative metabolites and similar fatty acid biosynthesis compared to healthy liver. Increased tumoral expression of glycolytic and hypoxia signaling pathway mRNAs was associated with decreased survival in HCC patients. In conclusion, HCC cells can rapidly alter their metabolism according to their environment and switch to the use of glucose through aerobic glycolysis to sustain their tumorigenicity and proliferative ability. Therefore, cancer metabolic reprogramming could be essential for the tumorigenicity of HCC cells during cancer initiation and invasion.

## INTRODUCTION

The ability of cancer to grow and proliferate in harsh environments has been a subject of interest for several decades [1, 2]. Since the demonstration that aerobic glycolysis took place in cancer cells by Otto Warburg [3], several important discoveries on the metabolic pathways of cancer cells have led to a better overall understanding of their ability to proliferate and adapt to their microenvironment [4]. Within a fluctuating microenvironment, such as in a low oxygen environment or with limited access to glucose, tumor cells will use other metabolic pathways such as the glutamine [5], lactate [6, 7] or fatty acid pathway [8] to overcome the low nutrient availability and generate energy essential to sustain their proliferation and survival. This metabolic reprogramming has been observed in several tissues including breast [9], lung [10] and liver cancer [11].

Hepatocellular carcinoma (HCC) is the fifth most common cancer worldwide and the third most lethal one [12]. Primary liver cancer develops in the vast majority of cases on a cirrhotic background [13]. Liver cirrhosis is characterized by an extensive modifications of hepatic lobular architecture with increased formation and deposition of extracellular matrix, eventually leading to a decrease in liver function [14]. These changes could significantly alter the microenvironment and metabolism of HCC tumors.

The liver is central to the homeostasis of global metabolism [15]. Among its many functions, it is involved in the metabolism of carbohydrates with gluconeogenesis, glycogenolysis and glycogenesis and in the metabolism of lipids with the synthesis and oxidation of triglycerides (TG) and fatty acids, the synthesis and transformation of cholesterol into bile acids, and in lipoprotein synthesis [16, 17]. All these metabolic functions are performed by hepatocytes which represent approximately 70% of the total number of hepatic cells and 90% of its mass [18]. The intrinsically high metabolic activity displayed by hepatocytes under physiological conditions hinders our ability to detect liver tumor foci by positron emission tomography (PET) since both neoplastic and healthy hepatocytes consume significant amounts of glucose [19]. Thus, studies on HCC and the metabolism of cancer cells are critical if we are to develop new carbohydrate tracers to better discriminate tumor cells from healthy liver cells. Fluctuations that occur within the environment of HCC cells could also impact their functional phenotype including when these cells are cultured *in vitro.* HCC cells can adapt to fluctuating conditions in the complex physiological conditions found in the normal liver such as within metabolic zonation where different oxygen tension gradients take place [20]. Therefore, metabolic reprogramming of HCC cells could be key to their tumorigenicity and the aggressiveness of HCC.

Metabolomics has made great strides in recent years especially in the identification of biomarkers that correlate with specific diseases or environmental exposure [21]. Moreover, metabolomics has recently been used to identify specific alterations in pathways in an effort to identify putative mechanisms that underlie various physiological conditions including several diseases [21]. Metabolites can be mapped and analyzed within metabolic pathways to link metabolites together and target specific pathways for studies [21].

Herein, using a metabolomic approach combined with functional *in vitro* and *in vivo* assays, we identified a metabolic signature associated with increased tumorigenicity in HCC cells and increased mortality in patients with HCC. We found that HCC Dt81Hepa1-6 cells, a highly tumorigenic derivative of Hepa1-6 cells [22], displayed increased metabolic plasticity *in vitro* and *in vivo* compared to primary hepatocytes or healthy liver tissues respectively. Their tumorigenicity was associated with increased levels of specific aerobic glycolysis metabolites and an increased ability to uptake glucose. This increased glucose uptake stemmed from an increased expression of glucose transporters. These HCC cells also showed increased expression of key glycolytic enzymes, enhanced levels of highly energetic metabolites and reduced levels of antioxidative stress-related metabolites. The functional phenotype of these HCC cells, unlike healthy hepatocytes, could rapidly change depending on their microenvironment; whereas these cells had a very active fatty acid synthesis *in vitro*, this was not apparent *in vivo* where extremely efficient aerobic glycolysis took place. Finally, HCC patients with high tumoral expression of glycolytic and hypoxia signaling pathway genes have a significantly decreased overall survival compared to patients with low expression of these genes. These observations suggest that metabolic reprogramming could be a driving factor for the tumorigenicity of these cells and critical for cancer stem cells, particularly during HCC initiation and invasion.

## RESULTS

### Metabolomics reveals that Dt81Hepa1-6 cell tumorigenicity stems from enhanced aerobic glycolysis and glucose utilization

**Figure 1:**
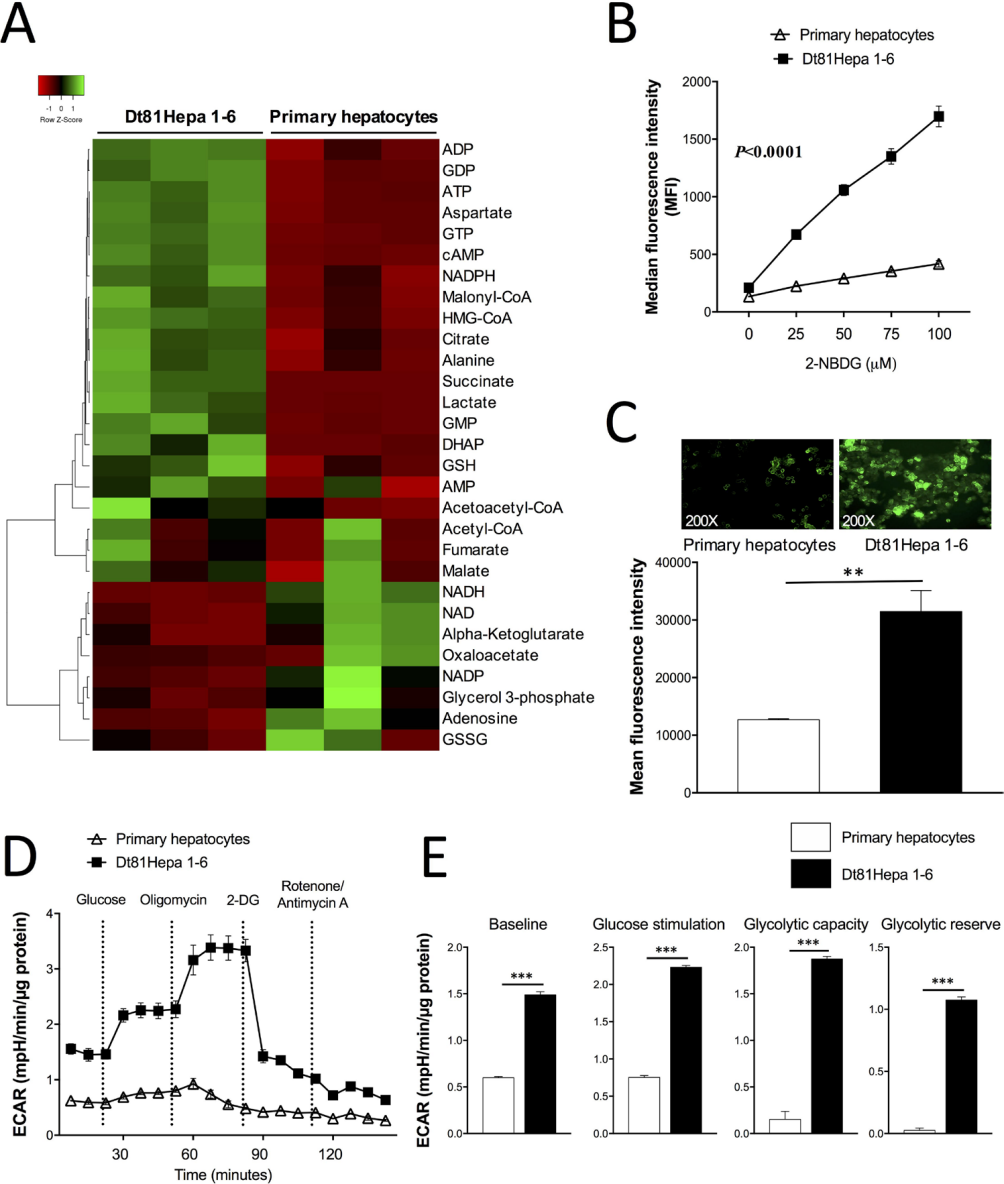
Metabolomic and glucose metabolism analysis of Dt81Hepa1-6 cells (**A**) Heatmap depicting the metabolomic analysis of 29 metabolites in Dt81Hepa1-6 cells and primary hepatocytes both cultured in 25 mM glucose for 48 hrs. (**B**) Median fluorescence intensity analysis of glucose uptake with increasing doses of glucose fluorescent analog 2-NBDG [0–100 mM] in glucose-free DMEM. (**C**) Fluorescent signal quantification and representative microphotographs of 2-NBDG-labeled primary hepatocytes and Dt81Hepa1-6 cells [50 mM of 2-NBDG]. (**D**–**E**) Extracellular acidification rate (ECAR) measurements using Seahorse XF24 Extracellular Flux analyzer. Primary hepatocytes and Dt81Hepa1-6 cells were cultured in 25 mM glucose DMEM for 48 hrs. Glycolytic capacity and glycolytic reserve were calculated based on the increase in ECAR after injection of oligomycin. Values are ± SEM of at least 3 independent experiments. (^**^P < 0.01, ^***^P < 0.001).

To identify the metabolic adaptations associated with the observed enhanced tumorigenicity of Dt81Hepa1-6 cells, we characterized the metabolic profile of Dt81Hepa1-6 and primary hepatocytes. Metabolomic analysis showed a clearly different profile for Dt81Hepa1-6 cells compared to primary hepatocytes. Heatmap analysis revealed that key aerobic glycolysis metabolites such as dihydroxyacetone phosphate (DHAP), lactate, ATP and GTP were increased in Dt81Hepa1-6 cells when compared to primary hepatocytes (Figure 1A). To assess the functional relevance of these observations, glucose uptake was measured using 2-NBDG, a fluorescent glucose analog. With increasing doses of 2-NBDG, Dt81Hepa1-6 consistently showed a higher capacity to uptake glucose in comparison to primary hepatocytes (*P* < 0.0001) (Figure 1B). Similarly, quantification of fluorescent images of cells exposed to a fixed dose of 2-NBDG revealed a greater glucose avidity by Dt81Hepa1-6 cells (*P* < 0.01) (Figure 1C).

To confirm that these HCC cells depend on the *Warburg effect* to drive their tumorigenicity, we studied glycolysis in both cell types using extracellular flux analysis (Seahorse XF24). Measurement of the extracellular acidification rate (ECAR) demonstrated a significantly higher glycolytic activity by Dt81Hepa1-6 cells compared to primary hepatocytes in culture for 48 hrs (Figure 1D). This analysis revealed that Dt81Hepa1-6 cells showed a higher level of non-glycolytic acidification baseline activity, an increased response to extracellular glucose, a higher glycolytic capacity as well as a higher glycolytic reserve (*P* < 0.001) (Figure 1E). Thus, Dt81Hepa1-6 cell tumorigenicity seems to be intimately linked to tumoral aerobic glycolysis. To ensure that the *in vitro* culture of primary hepatocytes for 48 hrs did not affect their glycolytic profile [23], we also compared the glycolytic phenotype of freshly isolated primary hepatocytes to that of hepatocytes cultured for 48 hrs (Supplementary Figure 1). Freshly isolated and primary cells cultured for 48 hrs demonstrated a very similar glycolytic profile (Supplementary Figure 1).

### The higher glycolytic activity of Dt81Hepa1-6 is associated with a rearrangement in glucose transporters at the cell surface

**Figure 2:**
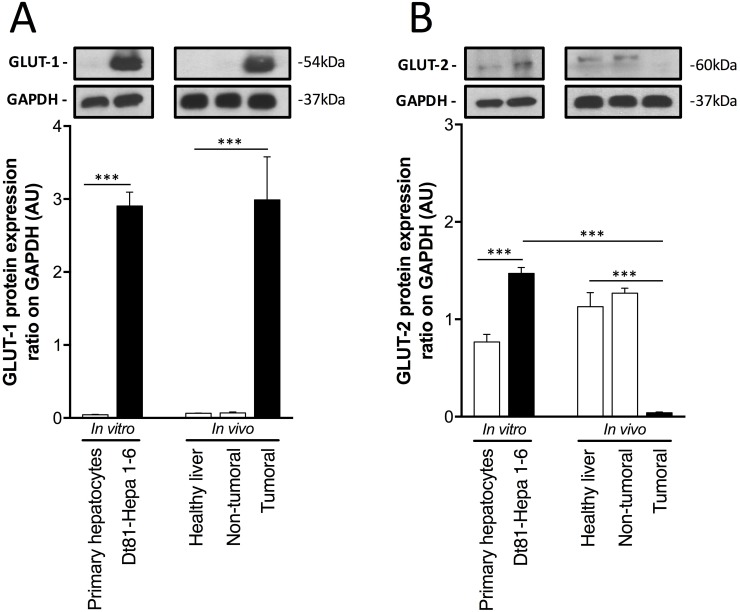
Increased glucose uptake by Dt81Hepa1-6 is mediated by a rearrangement of glucose transporters (**A**–**B**) Protein levels of Glucose transporter 1 (GLUT-1) and Glucose transporter 2 (GLUT-2) in primary hepatocytes and Dt81Hepa1-6 cells after 48 hrs incubation in 25 mM glucose DMEM (*In vitro*) and in healthy liver, non-tumoral and tumoral liver specimens (*In vivo*). Values are ± SEM of at least 3 independent experiments. (^***^P < 0.001).

Given the capacity of Dt81Hepa1-6 cells to uptake large amounts of extracellular glucose and use this additional glucose for glycolytic activity, we evaluated the expression level of glucose transporters *in vitro* and *in vivo* to ascertain if the enhanced tumorigenicity and glycolytic activity of Dt81Hepa1-6 *in vivo* originated from a rearrangement in glucose transporters at the cell surface. First, to ensure the validity of our *in vivo* tumoral, non-tumoral and healthy liver samples, histological analysis was performed and the expression levels of *Alfa-fetoprotein (AFP)* and *Epithelial cell adhesion molecule (Epcam)* were measured in these samples (Supplementary Figure 2). Histology confirmed the status of the liver samples and only Dt81Hepa1-6-derived tumors showed expression of *AFP* and *Epcam* (*P* < 0.001) (Supplementary Figure 2). Expression of glucose transporter GLUT-1 was increased exclusively in tumorigenic Dt81Hepa1-6 cells and Dt81Hepa1-6-derived tumors (*P* < 0.001) (Figure 2A). However, the expression of the GLUT-2 was differentially modulated according to the cell localization/environment (*in vitro* or *in vivo*) (Figure 2B). *In vitro,* Dt81Hepa1-6 cells displayed high protein levels of GLUT-2 compared to primary hepatocytes (*P* < 0.001) whereas *in vivo,* Dt81Hepa1-6-derived tumors showed a significantly lower GLUT-2 protein expression level when compared to healthy and peri-tumoral liver specimens (*P* < 0.001) (Figure 2B).

### *In vivo* metabolic adaptation by tumorigenic Dt81Hepa1-6 leads to increased expression of aerobic glycolysis genes

**Figure 3:**
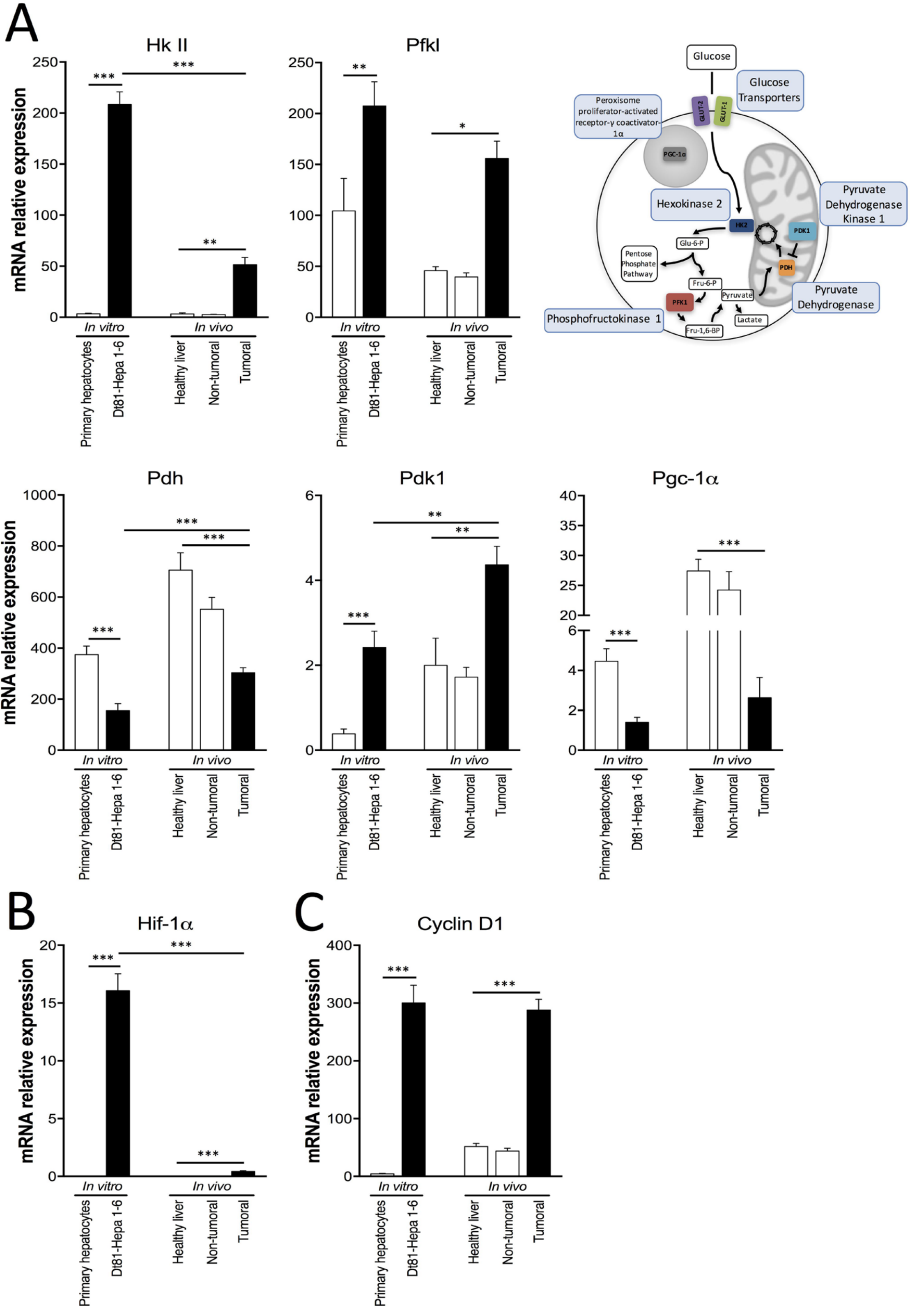
Expression of glycolysis-related genes by Dt81Hepa1-6 cells and Dt81Hepa1-6-derived tumors mRNA gene expression of (**A**) *Hexokinase II* (*Hk II*), *Phosphofructokinase liver* (*Pfkl*), *Pyruvate dehydrogenase* (*Pdh*), *Pyruvate dehydrogenase kinase 1* (*Pdk1*), *Peroxisome proliferator-activated receptor gamma coactivator 1-alpha* (*Pgc-1α*), (**B**) *Hypoxia inducible factor-1alpha* (*Hif-1α*) and (**C**) *Cyclin D1*, in primary hepatocytes and Dt81Hepa1-6 cells after a 48 hrs incubation in 25 mM glucose DMEM (*In vitro*) and in healthy liver, non-tumoral and tumoral liver specimens (*In vivo*). Values are ± SEM of at least 3 independent experiments. (^*^P < 0.05, ^**^P < 0.01, ^***^P < 0.001).

To assess the metabolic flexibility of tumorigenic Dt81Hepa1-6 cells, the activity of aerobic glycolysis was evaluated and compared with normal hepatocytes and between *in vitro* and *in vivo* conditions. mRNA quantification of several glycolytic genes was performed. First, both Dt81Hepa1-6 cells and Dt81Hepa1-6-derived tumors displayed increased mRNA levels of *Hexokinase II* (*Hk II*) (*P* < 0.01), *Phosphofructokinase liver* (*Pfkl*) (*P* < 0.05), *Pyruvate dehydrogenase kinase 1* (*Pdk1*) (*P* < 0.01) and decreased mRNA expression levels of *Pyruvate dehydrogenase* (*Pdh*) (*P* < 0.001) and *Peroxisome proliferator-activated receptor gamma coactivator 1-alpha* (*Pgc-1α*) (*P* < 0.001) (Figure 3A), in comparison to primary hepatocytes and healthy and peri-tumoral liver specimens. Interestingly, Dt81Hepa1-6-derived tumors displayed lower *Hk II* expression (*P* < 0.001) but higher *Pdh* and *Pdk1* expressions in comparison to Dt81Hepa1-6 cells *in vitro* (*P* < 0.01) (Figure 3A). Dt81Hepa1-6 cells and Dt81Hepa1-6-derived tumors also showed higher expression levels of *Hypoxia-inducible factor-1alpha* (*Hif-1α*) and *Cyclin D1* compared to primary hepatocytes and healthy and peri-tumoral liver specimens (*P* < 0.001) (Figure 3B–3C). While the *Hif-1α* mRNA expression *in vivo* was lower in tumors than *in vitro* (*P* < 0.001) (Figure 3B), the expression of *Cyclin D1* was increased both *in vitro* and *in vivo* in Dt81Hepa1-6 cells (Figure 3C).

### Dt81Hepa1-6-derived tumors display an increased energetic profile compared to healthy and peri-tumoral liver tissue

**Figure 4:**
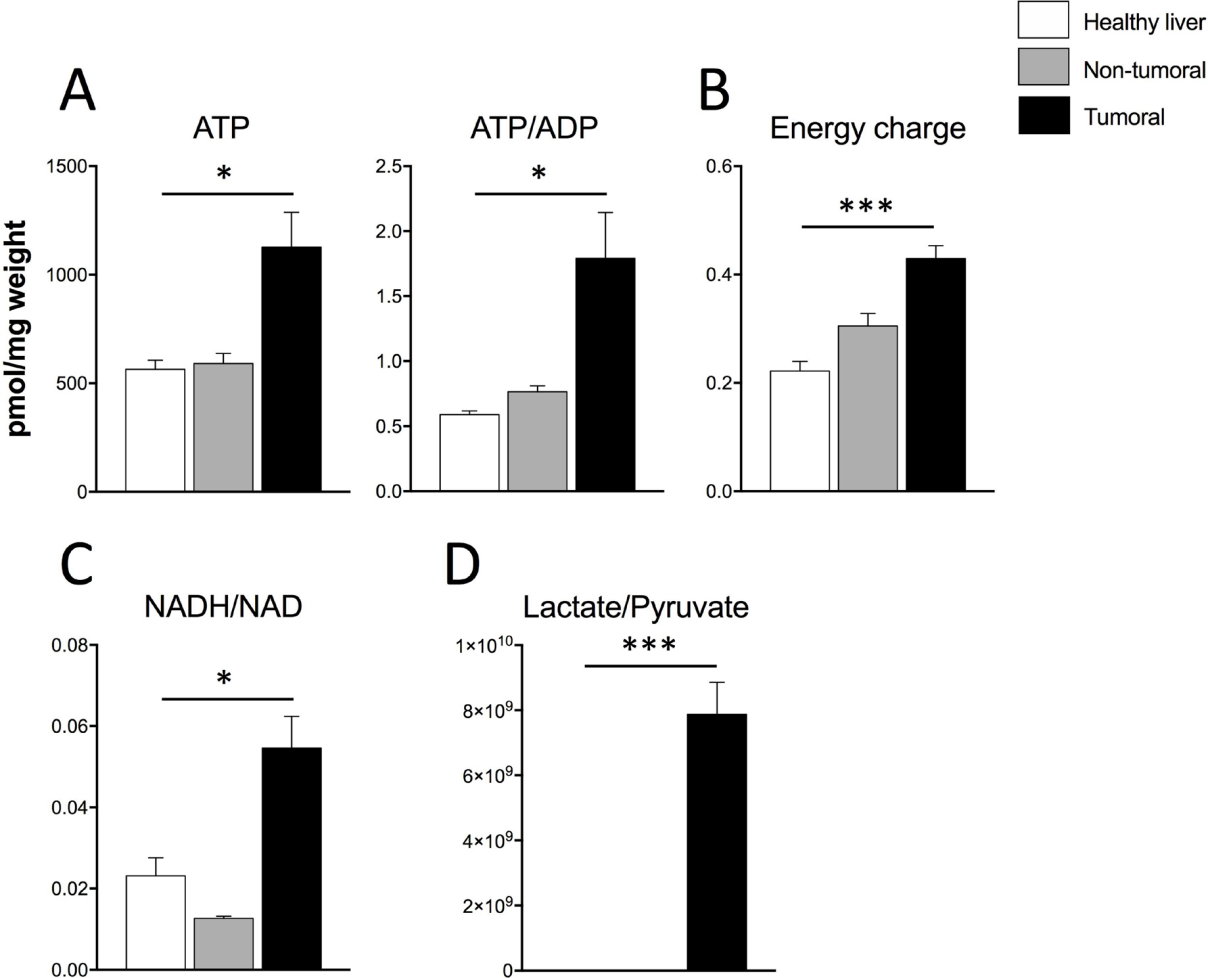
Energetic profile of Dt81Hepa1-6-derived tumors greatly differs from that of healthy liver and non-tumoral samples (**A**) Evaluation of total intracellular ATP and ATP/ADP ratio, (**B**) calculated Energy charge values, (**C**) NADH/NAD and (**D**) Lactate/Pyruvate ratios in healthy liver, non-tumoral and tumoral liver specimens. Values are ± SEM of at least 3 independent experiments. (^*^P < 0.05, ^***^P < 0.001).

If Dt81Hepa1-6-derived tumors display a *Warburg effect* and an increased ability to proliferate *in vivo,* this should translate into an increased capacity to produce energy. Therefore, we quantified three major cellular energetic metabolites: AMP, ADP and ATP. Dt81Hepa1-6-derived tumors displayed higher ATP levels and a higher ATP/ADP ratio when compared to healthy and peri-tumoral liver specimens (*P* < 0.05) (Figure 4A). On the other hand, normal healthy liver had significantly higher levels of AMP and ADP (*P* < 0.05) (Supplementary Figure 3). Dt81Hepa1-6-derived tumors also displayed a higher energy charge level when compared to healthy and peri-tumoral liver specimens (*P* < 0.001) (Figure 4B). Finally, elevated NADH/NAD and Lactate/Pyruvate ratios were found only in tumoral tissue (*P* < 0.05 and *P* < 0.001, respectively) (Figure 4C–4D).

### Dt81Hepa1-6-derived tumors and peri-tumoral liver specimens have lower levels of antioxidative stress-related metabolites

**Figure 5:**
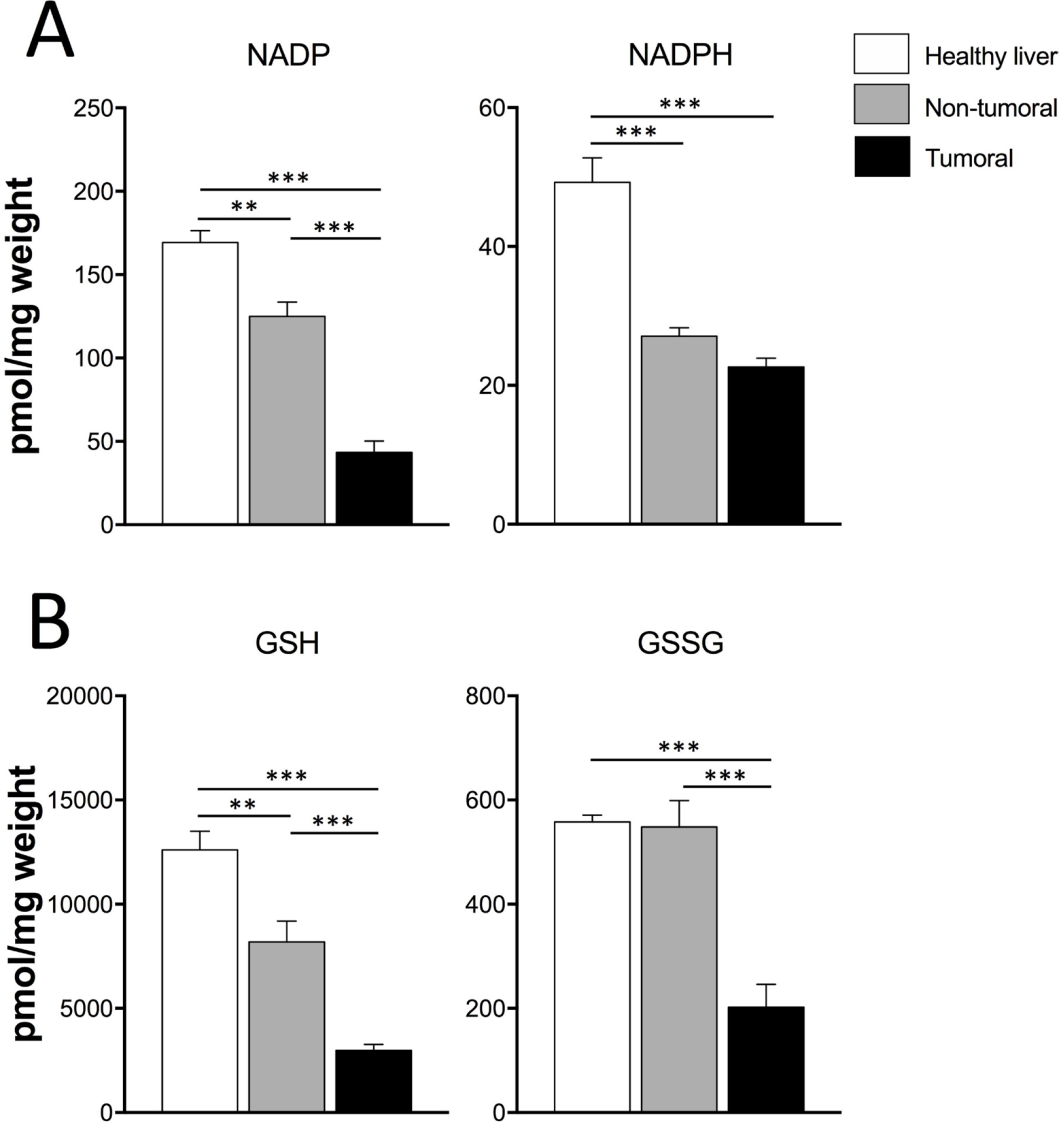
Dt81Hepa1-6-derived tumors show lower contents of antioxidative-related metabolites (**A**–**B**) Total intracellular NADP, NADPH and GSH, GSSG levels in healthy liver, non-tumoral and tumoral liver specimens. Values are ± SEM of at least 3 independent experiments. (^**^P < 0.01, ^***^P < 0.001).

Hypoxia, oxidative stress and reactive oxygen species (ROS) can influence metabolic reprogramming of cancer cells and play a fundamental role in tumor invasion and maintenance [24, 25]. Therefore, we evaluated the levels of antioxidative stress-related metabolites in our cells and tissue specimens. First, the degree of hypoxia was assessed *in vivo* by measurement of HIF-1α protein level. This analysis found high HIF-1*α* protein levels only in tumoral tissues (*P* < 0.01) (Supplementary Figure 4) corroborating the increased *Hif-1a* mRNA observed in tumors (*P* < 0.001) (Figure 3B). Dt81Hepa1-6-derived tumors had lower NADP (*P* < 0.001), NADPH (*P* < 0.001), GSH (*P* < 0.001) and GSSG (*P* < 0.001) contents in comparison to healthy liver specimens (Figure 5A–5B). Interestingly, similar differences were found between non-tumoral samples and healthy liver specimens including NADP (*P* < 0.01), NADPH (*P* < 0.001) and GSH levels (*P* < 0.01) (Figure 5A–5B). Only GSSG levels were similar between non-tumoral and control healthy tissues.

### Role of fatty acid metabolism in Dt81Hepa1-6 cells tumorigenicity and in the survival of HCC patients

**Figure 6:**
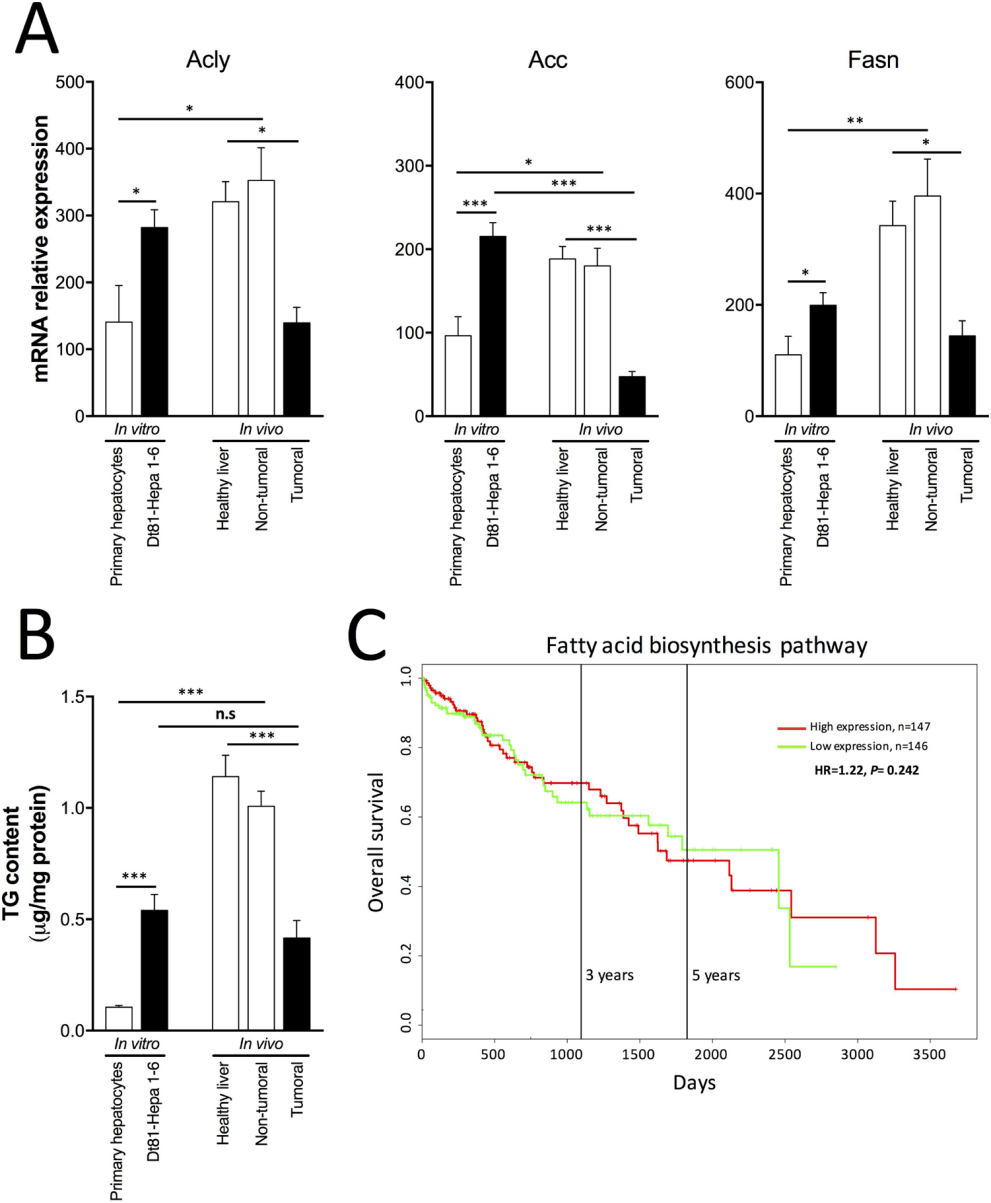
Fatty acid biosynthesis in HCC cells (**A**) mRNA relative gene expression of *ATP citrate lyase* (*Acly*), *Acetyl-CoA carboxylase* (*Acc*), *Fatty acid synthase* (*Fasn*), (**B**) assessment of triglyceride (TG) content, respectively in primary hepatocytes and Dt81Hepa1-6 cells after a 48 hrs incubation in 25 mM glucose DMEM (*In vitro*) and in healthy liver, non-tumoral and tumoral liver specimens (*In vivo*). (**C**) Kaplan–Meier (KM) plots of Overall survival probability of HCC cancer patients (TCGA data). Patients have been stratified into high (red lines) or low (green lines) expression-based ‘risk-groups’ by their mean of median transcript-expressions of fatty acid biosynthesis related genes. The patient follow-up is indicated in days. Respective Log-rank test *p*-values and Hazard Ratio (HR) are shown. The numbers of patients for each group are indicated below the respective KM plots. Studied genes are described in Supplementary Table 2.

Fatty acids derived from glucose metabolism can be pivotal for cancer cell growth and survival. Hence, their rate of biosynthesis could impact the tumorigenicity of HCC cells *in vitro* and *in vivo*. Therefore, to evaluate if the fatty acid biosynthesis pathway is involved in Dt81Hepa1-6 tumorigenicity, we quantified the genes and metabolites involved in this biosynthetic process. When cultured *in vitro* at the highest glucose concentration, Dt81Hepa1-6 cells displayed higher mRNA expression levels of *ATP citrate lyase* (*Acly*) (*P* < 0.05), *Acetyl-CoA carboxylase* (*Acc*) (*P* < 0.001) and *Fatty acid synthase* (*Fasn*) (*P* < 0.05) in comparison to primary hepatocytes (Figure 6A). Analysis of TG content also showed increased levels only in cultured Dt81Hepa1-6 cells (*P* < 0.001) (Figure 6B). Interestingly, Dt81Hepa1-6-derived tumors displayed lower mRNA expression levels of *Acly* (*P* < 0.05), *Acc* (*P* < 0.001) and *Fasn* (*P* < 0.05) as well as decreased TG content (*P* < 0.001) when compared to healthy control and non-tumoral liver specimens (Figure 6A–6B). The only difference observed between *in vitro* Dt81Hepa1-6 cells and *in vivo* Dt81Hepa1-6-derived tumors was a decreased mRNA expression level of *Acc* in tumors (*P* < 0.001) (Figure 6A). No significant differences in TG content were found between *in vitro* and *in vivo* Dt81Hepa1-6 cells (Figure 6B). Healthy control and non-tumoral liver specimens displayed higher mRNA levels of *Acly* (*P* < 0.05), *Acc* (*P* < 0.05) and *Fasn* (*P* < 0.01) and increased TG content (*P* < 0.001) when compared to primary hepatocytes (Figure 6A–6B). Finally, to evaluate if fatty acid biosynthesis could correlate with increased tumorigenicity and mortality in patients with HCC, we examined the association between the levels of tumoral mRNA expressions of 13 genes involved in this anabolic pathway (listed in Supplementary Table 2) with patients’ survival using publicly-available expression datasets from HCC patients [26, 27]. No significant difference was found in overall survival of patients with high- and low-level expressing tumors (*Ptges3, Fads1, Ptgs1, Mcat, Fads2, Cd74, Brca1, Mif, Oxsm, Ptgds, Lta4h, Hpgd* and *Degs1*) (Figure 6C) ((*n* = 293) *P* = 0.242).

### Increased expression of glycolytic and hypoxia-induced response genes by tumors from patients with HCC correlates with poor survival

**Figure 7:**
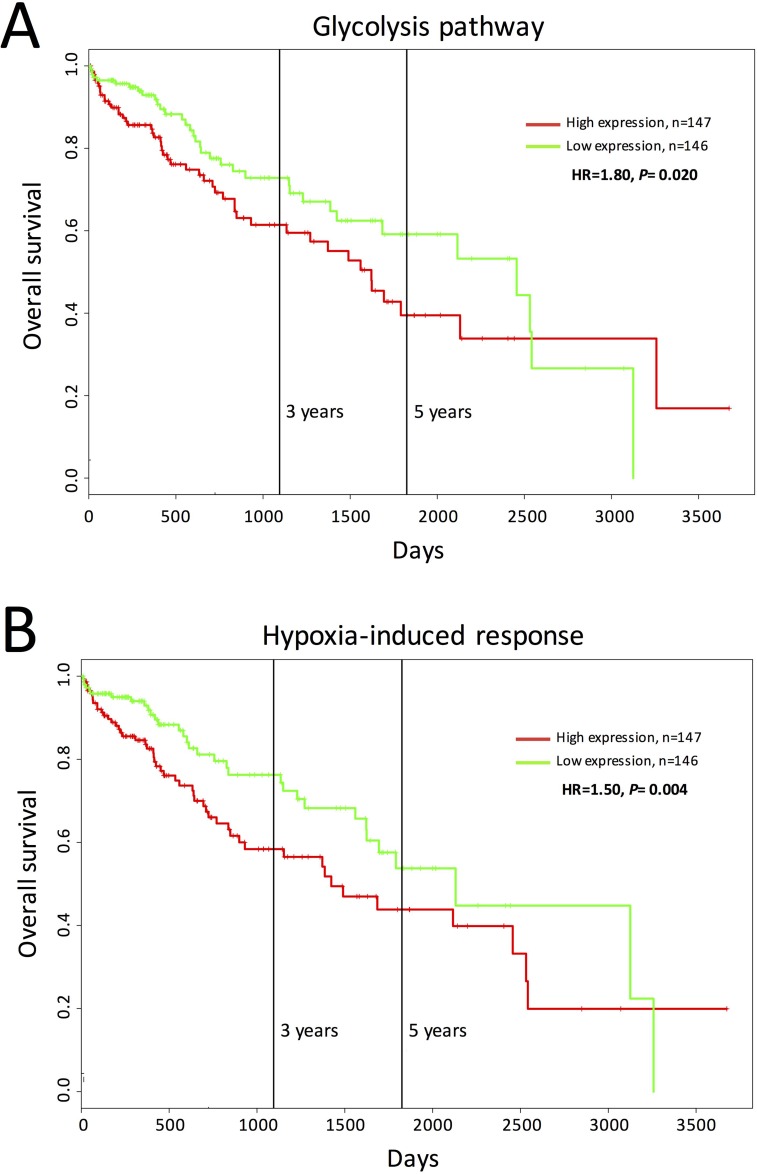
High expressions of glycolysis and hypoxia-induced response genes are associated with poor prognosis in HCC patients Kaplan–Meier (KM) plots of Overall survival probability of HCC cancer patients (TCGA data). Patients have been stratified into high (red lines) or low (green lines) expression-based ‘risk-groups’ by their mean of median transcript-expressions of (**A**) glycolytic and (**B**) hypoxia-induced response genes. Patient follow-up is indicated in days. Respective Log-rank test *p*-values and Hazard Ratio (HR) are shown. The numbers of patients for each group are indicated below the respective KM plots. Studied genes are described in Supplementary Table 2.

The previous results suggest that the tumorigenicity of Dt81Hepa1-6-derived tumors *in vivo* stems from an increased glycolytic activity and a higher hypoxia-induced response. To assess if activation of these biological pathways is associated with increased tumorigenicity and mortality in patients with HCC, we examined the association between the levels of glycolytic and hypoxia signaling mRNA expressions (27 genes for each biological pathway, listed in Supplementary Table 2) and patient survival using the PROGgene V2 expression datasets from HCC patients [26, 27]. HCC patients with tumors displaying higher levels of glycolytic (*Eno1, Eno2, Eno3, Aldoa, Aldob, Aldoc, Gapdh, Gapdhs, Gpi, Pfkfb1, Pfkfb2, Pfkfb3, Pfkfb4, Pfkl, Pfkm, Pfkp, Pgam1, Pgam2, Pgk1, Pklr, Pkm2, Ppp2ca, Ppp2cb, Ppp2r1a*, *Ppp2r1b, Ppp2r5d* and Tpi1) and hypoxia signaling (*Cldn3, Pdia2, Arnt2, Pml, Egln2, Bnip3, Egln1, Tgfb2, Alas2, Plod1, Plod2, Cxcr4, Ang, Chrna4, Cd24, Mt3, Epas1, Nf1, Crebbp, Smad4, Smad3, Hsp90b1, Ep300, Hif1a, Vegfa, Narfl* and *Chrnb2*) mRNA were shown to have a significantly decreased overall survival when compared to patients with lower levels of expressing tumors (Figure 7) ((*n* = 293) respectively *P* = 0.020 and *P* = 0.004).

## DISCUSSION

The first tumor cell-specific metabolic alteration was described by Otto Warburg: he showed an increase in the activity of glycolysis that was maintained in conditions of high oxygen tension [3]. Since then, metabolic reprogramming has often been described as a hallmark of cancer cell transformation [1, 2]. Owing to the heterogeneous distribution of crucial nutrients (in particular oxygen and glucose) and the ability of tumor cells to adapt to a nutritionally-restricted microenvironment, the question remains as to how cancer cells can cope with these stresses and still maintain growth and proliferation. Given the heterogeneous nature of the HCC tumoral microenvironment, adaptive mechanisms to maintain energy production and metabolic homeostasis must exist in HCC cells if proliferation is to be maintained. These cells have to adapt metabolically to the changing, nutrient-restricted environment characteristic of liver fibrosis and cirrhosis to maintain their tumorigenic potential. Understanding how the metabolism of HCC cells is changed according to different microenvironments could lead to the identification of novel putative HCC-specific metabolites. This could allow for the identification of novel tracers that would help discriminate between healthy and tumoral liver tissues [19, 28].

Using a metabolomic approach, we found that Dt81Hepa1-6 HCC cells behave metabolically very differently than primary hepatocytes under identical *in vitro* conditions. This suggests that these cells have developed metabolic strategies in order to strive. The metabolite signature of these cells suggests that they have the capacity to modify their bioenergetic and fatty acids metabolism as well as their glycolytic activity.

To understand the origin of the increase in glycolytic activity, we studied glucose metabolism from its entry into the cells to the production of energetic metabolites. Dt81Hepa1-6 cells displayed an enhanced ability to efficiently uptake extracellular glucose and a greater avidity for it compared to primary hepatocytes under identical conditions. The entry of glucose in cells is mediated by transporters located at the plasma membrane and their expression level correlates with cancer aerobic glycolysis [29, 30]. GLUT-1 and GLUT-2 are the two main glucose transporters in the liver [31]. Dt81Hepa1-6 HCC cells showed significantly higher levels of GLUT-1 and GLUT-2 *in vitro* in comparison to normal hepatocytes. *In vivo*, Dt81Hepa1-6-derived tumors also had higher levels of GLUT-1 but decreased levels of GLUT-2 in comparison to normal liver. Several observations could explain the decreased expression of GLUT-2 by tumoral liver specimens. *In vivo*, extracellular glucose supply can fluctuate; in order to optimize glucose uptake, neoplastic cells have the ability to use glucose transporters differently such as increasing expression of GLUT-1 [32]. GLUT-1 offers a steady influx of glucose to cells in contrast to GLUT-2 that will drive glucose entry preferentially in conditions of hyperglycemia. In HCC, increased expression of GLUT-1 and reduced expression of GLUT-2 have been shown to correlate with an aggressive phenotype [11, 33]. This decrease in GLUT-2 is likely the result of a loss of differentiation since hepatocytes are characterized by high GLUT-2 expression *in vivo* [34]. Therefore, the change between *in vitro* conditions where GLUT-1 and GLUT-2 expression are tightly regulated and the physiologically more complex *in vivo* environment where tumor cells need to adopt new metabolic strategies, would lead to a rearrangement of glucose transporters expressed at the cell surface in order to 1) efficiently uptake glucose and 2) maintain a high tumorigenic potential through sustained glycolysis.

Evaluation of glycolysis using extracellular flux analysis also showed an increased glycolytic activity by Dt81Hepa1-6 with an increased glycolytic capacity and glycolytic reserve suggesting that the increased uptake of glucose is coupled with an increased in glucose metabolism. Evaluation of mRNA expression of several glycolytic genes (*Hk II*, *Pfkl*, *Pdh*, *Pdk1*, *Pgc-1α*) showed that their levels were significantly increased when compared to primary hepatocytes or healthy liver. Interestingly, Dt81Hepa1-6-derived tumors had reduced *Hk II* expression and increased *Pdh* and *Pdk1* expressions in comparison to Dt81Hepa1-6 cells in culture suggesting that the expression of these enzymes is also modulated according to the environment. These changes could occur in order to sustain the production of energetic metabolites despite changes in the availability of nutrients. We observed a significant increase in the ATP content, ATP/ADP ratio and energy charge level in tumoral liver samples suggesting that the modulations we observed in the expression of glycolytic enzymes did translate into improved energy production.

In recent years, a growing body of evidence points towards a critical function for mitochondria in neoplastic cells that could combine with the *Warburg effect* during cancer metabolic reprogramming [35]. In contrast to the original Warburg hypothesis, many cancers maintain functional mitochondria and flux through the electron transport chain. This helps provide optimal levels of ATP, essential for their survival [36, 37]. Our observations suggest, based on the increased NADH/NAD and Lactate/Pyruvate ratios, and increased gene expressions of *Pdh* and *Pdk1*, that Dt81Hepa1-6 cells shift away from mitochondrial activity and direct their metabolic activity toward aerobic glycolysis.

Cancer cells can also rely on the activation of alternative metabolic pathways to maintain their proliferative capacities, such as the synthesis and use of fatty acids. These compounds are essential for the synthesis of new plasma membranes, for lipid-based post-translational modification of proteins, to promote DNA replication and induce rapid cell division but also to prevent cell death through the activation of anti-apoptotic proteins [38, 39]. Thus, the ability of cancer cells to synthesize fatty acids represents a critical aspect of HCC cell tumorigenicity. *In vitro*, Dt81Hepa1-6 cells displayed an increased ability to synthesize fatty acids, as evidenced by their increased expression of fatty acid-related genes and TG content. However, *in vivo*, this was not observed when healthy and non-tumoral tissues were compared. In addition, the expression of fatty acid biosynthesis gene by tumors from patients with HCC was not associated with overall patient survival. Therefore, the constraints exerted by the microenvironment would lead HCC cells to adapt and use the most metabolically effective pathways to maintain their tumorigenicity. In the case of Dt81Hepa1-6-derived tumors, they display a metabolic adaptation for the preferential use of glucose for aerobic glycolysis and ATP production over the synthesis of fatty acids.

Interestingly, we observed the same metabolic change of glycolytic activity in human HepG2 *vs* Huh7 HCC cells that we noticed in mice Dt81Hepa1-6 HCC cells: the increased glycolytic activity was only observed in highly tumorigenic Dt81Hepa1-6 and Huh7 HCC cells [40].

In conjunction with the nucleus, mitochondria are able to transcribe and translate genes encoding components of the electron transport chain via the production of ROS [41]. However, mitochondrial signaling can be altered in cancer cells and an increase in ROS production has been described in tumor cells [42, 43]. Mitochondrial-generated ROS have been proposed to behave as initiation factors for many important signaling pathways in which a mitochondrial signal induces changes in nuclear gene expression, thus influencing overall cellular function. Therefore, the type and amount of ROS found within each type of neoplastic cells for a given microenvironment could have a significant influence on its tumorigenicity. *In vivo*, Dt81Hepa1-6-derived tumors displayed lower contents of redox metabolites NADP, NADPH, GSH, and GSSG. Interestingly, some significant variations were also noticed between non-tumoral and healthy liver samples. These differences could be explained by the non-homogeneous ROS distribution from tumors to non-tumoral adjacent tissues, highlighting the effect of tumoral cells on surrounding healthy tissue [44–46]. This deleterious effect could lead to a metabolic instrumentalization of non-cancerous cells to generate energy in favor of neoplastic cells, but also to the gradual transformation of healthy cells into tumoral cells [45–47]. This emphasizes the notion of intratumoral heterogeneity and has notably been observed in breast cancer, giving rise to the terminology of *Reverse Warburg effect* [48, 49].

Tumor hypoxia has been shown to be involved in tumorigenicity and malignancy since it can push cancer cells into metabolic reprogramming. However, the assessment of tumor hypoxia used to be based on direct p0_2_ tissue measurements which had important disadvantages including the invasive nature of the procedure and the technical difficulty of accessing tumors [50]. Recently, novel biomarkers of hypoxia such as HIF-1α and GLUT-1 have been described [51, 52]. Since HIF-1α is absent in *in vitro*-cultured Dt81Hepa1-6 cells when maintained under normoxic conditions (data not shown), the elevated HIF-1α and GLUT-1 levels observed *in vivo* indicate that these cells are in a hypoxic environment with low O_2_ tension and that this environment could be responsible for the metabolic changes observed in these cells *in vivo.* In addition, tumor progression, angiogenesis and anaerobic metabolism enable cancer cells to survive under hypoxia as Hk II was shown to be selectively regulated through a HIF-1α-dependent mechanism [53, 54]. Thus, cells that could respond effectively to these environmental cues could rapidly adapt to sustain their growth and tumorigenicity.

Therefore, the increased tumorigenicity of Dt81Hepa1-6-derived HCC tumors would stem from an increased glycolytic activity and an increased hypoxia-induced response. Interestingly, we found that HCC patients with increased tumoral glycolytic and hypoxic mRNAs expressions have significantly decreased survival rate. This suggests that these metabolic pathways are directly linked with HCC tumor aggressiveness and therefore, could be targeted in order to reduce tumorigenicity and potentiate current therapies.

In conclusion, these results strongly suggest that HCC cells can rapidly adapt to their environment and adopt a metabolic strategy aimed at using glucose through aerobic glycolysis to sustain their energy requirements, tumorigenicity, and proliferative ability. These observations suggest that cancer metabolic reprogramming constitutes an essential factor for the tumorigenicity of these cells and could be critical for cancer stem cells, particularly during HCC initiation and invasion.

## MATERIALS AND METHODS

### Reagents

Dulbecco's Modified Eagle Medium (DMEM), Leibovitz's L-15 medium, Fetal Bovine Serum (FBS), penicillin/streptomycin, fluorescent glucose analog 2-[N-(7-nitrobenz-2-oxa-1,3-diaxol-4-yl) amino]-2- deoxyglucose (2-NBDG) and TRIZOL™ reagent were purchased from Invitrogen (Burlington, On, Canada). QuantiTect reverse transcription kit and QuantiTect SYBR Green PCR Kit were purchased from QIAGEN (Toronto, On, Canada). Type IV Collagenase was from Worthington-Biochemicals inc. (Lakewood, NJ). Unless stated otherwise, all other products were from Sigma-Aldrich (Oakville, On, Canada).

### Hepatocyte isolation

Hepatocytes were isolated from adult male C57BL/6 mice using the two-step collagenase perfusion method as previously described [55]. Briefly, under anesthesia, the peritoneal cavity was opened, and the liver was perfused *in situ* via the portal vein for 4 min at 37° C with calcium-magnesium(CM)-free HEPES buffer and for 7 min with CM-free HEPES buffer containing Type IV collagenase (35 mg/100 ml) and CaCl_2_ [10 mM]. Cells were used only if cell viability was above 80% as assessed by trypan blue exclusion. After three centrifugations (44 g for 2 min) in Leibovitz's L-15 media supplemented with 0, 2% bovine albumin, cells were seeded onto plastic Petri dishes. After cell attachment for 2 hrs, the medium was replaced by fresh medium supplemented with 10% FBS.

### Cell culture conditions

Authenticated Hepa1-6 murine hepatoma cell line was obtained from the American Type Culture Collection (Manassas, Virginia, USA). Dt81Hepa1-6 cell line was derived from Hepa1-6 cells through *in vivo* passage in C57BL/6 mice [22]. Primary hepatocytes and Dt81Hepa1-6 cells were maintained at 37° C and 5% CO_2_ and cultured in standard 25 mM glucose DMEM, supplemented with 10% FBS for 0 or 48 hrs following cell attachment (2 hrs for primary hepatocytes and overnight for Dt81Hepa1-6 cells). A concentration of 25 mM glucose was used since primary hepatocytes display important morphological alterations when maintained under lower glucose concentrations [23]. 25 mM glucose DMEM was used for both primary and Dt81Hepa1-6 cell culture to be able to compare each other under the same culture conditions. All culture media contained penicillin [100 units/ml] and streptomycin [100 μg/ml]. Cells were seeded at 0.026 M cells/cm^2^ for primary hepatocytes and 0.250 M cells/cm^2^ for Dt81Hepa1-6 to achieve 70% of cell confluence.

### Animals

Male C57BL/6 mice (20 g) were purchased from Charles River (Saint-Constant, Qc, Canada) and fed *ad libidum* with normal Chow. Animals were monitored daily for their appearance, state of hydration, behavior and clinical signs. Animals were sacrificed by exsanguination under anesthesia (induction with inhaled 4% Isoflurane and maintenance with inhaled 2% Isoflurane). All procedures were performed in accordance with Canadian Council on Animal Care and approved by the *Comité institutionnel de protection animale (CIPA) du CHUM*.

### Intrasplenic Dt81Hepa1-6 cell injection

The Dt81Hepa1-6 cell line was trypsinized and resuspended in a saline solution containing 0.25% albumin. An aliquot (200 μL) of 1M Dt81Hepa1-6 cells was loaded in 25 G syringes for intrasplenic injection. Under anesthesia, an abdominal incision was performed and the spleen was pulled out on a 37° C saline-soaked gauze. The syringe was mixed and the needle inserted in the spleen parenchyma and cells were slowly injected. When the spleen regained its bright red color, the needle was slowly drawn back and a droplet of Vetbound veterinary glue (3 M, London, On, Canada) applied. The spleen was put back into the abdominal cavity and the abdominal incision closed.

### *In vivo* characterization of Dt81Hepa1-6-derived tumors

For *in vivo* analyzes, intrahepatic tumors were obtained following intrasplenic injection of Dt81Hepa1-6 cells (1 M) in mice that were sacrificed 21 days later. Dt81Hepa1-6-derived tumors (Tumoral), neighboring normal liver parenchyma (Non-tumoral) were dissected, snap-frozen and then kept at −80° C until analysis. Additional healthy liver tissue (not subjected to any surgical procedure) were used as controls.

### Histological analysis

Formalin-fixed liver samples obtained at the time of sacrifice were set in paraffin blocks, sliced (4 μm sections) and stained with hematoxylin-phloxine-saffron by the Pathology Department of CHUM. Microphotographs were taken with a Carl-Zeiss Axioplan 2 microscope (Göttingen, Germany) at 10× magnifications using the Northern Eclipse 6.0 software (Empix Imaging, Mississauga, ON, Canada).

### Glucose uptake assay

Following 30 min of glucose starvation, primary hepatocytes and Dt81Hepa1-6 cells were incubated in glucose-free DMEM for 45 min in the presence of a fluorescent analog, 2-NBDG, at concentrations ranging from 0 to 100 μM. All subsequent steps were performed in the dark. The 2-NBDG reaction was stopped by washing cells with ice-cold phosphate-buffered saline (PBS). Glucose uptake was then quantified by measuring the fluorescent intensity of cells on a FACS BD LSRII flow cytometer (BD Biosciences, Mississauga, On, Canada). Data analysis was performed using FlowJo v10 (Tree Star, Ashland, Or, USA). Acquisition of fluorescent images was performed using a Leica Epifluorescence Microscope SP5 platform (Leica Microsystems, Richmond Hill, On, Canada). Quantitative analysis of 2-NBDG-labeled primary hepatocytes and Dt81Hepa1-6 cells was done using Fiji software (ImageJ, NIH, USA).

### qPCR gene expression analysis

mRNA was isolated with TRIZOL (Invitrogen (Burlington, On, Canada) according to the manufacturer guidelines. 250 ng of mRNA was subjected to reverse transcription using the QuantiTect Reverse Transcription Kit. Quantitative PCR amplifications were performed using the QuantiTect SYBR Green PCR Kit in a Rotor-Gene 3000 Real-Time Thermal Cycler (Corbett Research, Sydney, Australia). For each gene tested, 35 amplification cycles at 59° C (annealing) were used. The primer sequences are summarized in Supplementary Table 1. Relative gene expression was evaluated using 3 reference genes: *HPRT1*, *Ppia* and *H2afz* [56]. Relative gene expression was calculated using the delta-delta CT method [57].

### HPLC analysis

All metabolites described in this study were assessed using HPLC (Agilent 1200 HPLC system, Agilent technologies Canada Inc., Mississauga, On, Canada) by the Metabolomic Core Facility of CRCHUM. Metabolic measurements were done both on cells and liver specimens. Cells (after removal of cell culture medium) and liver samples were snap frozen in liquid nitrogen and kept at −80° C until HPLC analysis. HPLC peak areas were used for quantification of each metabolite. To normalize the metabolite relative quantification in primary hepatocytes and Dt81Hepa1-6 cells, total protein content was measured (Bradford protein assay [58]). For quantification of metabolites in liver specimens, the same amount of biological material (20 mg) was used for each of the samples tested. Energy Charge was calculated using this formula: ([ATP] + ½[ADP]) / ([ATP] + [ADP] + [AMP]) [23]. Graphic representation of the differential metabolite expression between primary hepatocytes and Dt81Hepa1-6 cells by heatmap was produced using Heatmapper [59].

### Seahorse XF24 extracellular flux analyzer

Primary hepatocytes and Dt81Hepa1-6 cells were seeded in the XF24 microplate in 25 mM glucose DMEM supplemented with 10% FBS for 0 and 48 hrs following cell attachment. According to manufacturer's recommended protocol, cell medium was replaced by conditional medium (culture medium without FBS and sodium bicarbonate) and incubated without CO_2_ for one hour before completion of sensor cartridge calibration. Extracellular acidification rate (ECAR) was measured in the Seahorse XF24 Flux analyzer (Agilent Technologies Canada Inc., Mississauga, On, Canada). Measurements were performed after injection of the following 4 compounds: 10 mM glucose, 2 μM oligomycin, 30 mM 2-deoxy-D-glucose (2-DG) and rotenone/antimycin, respectively at 1 and 2 μM. Glycolytic capacity and glycolytic reserve were calculated by measuring the increase in ECAR after injection of oligomycin [60]. Upon completion of the Seahorse XF24 Flux analysis, cells were lysed to calculate the protein concentration using the Bradford method. Results were normalized based on the total amount of proteins in each well.

### Western blotting

Cells were lysed in RIPA buffer containing phosphatase and protease inhibitors. Proteins were quantified in supernatants using the Bradford method. Samples were boiled during 5 min (except for GLUT-1 quantification) and then loaded (10 μg protein/well) onto 12% SDS-polyacrylamide gel electrophoresis, and SDS-PAGE migration was performed (1 hr, 150 V). Proteins were transferred to PVDF membranes (30 min, 25 V). Membranes were blocked in PBST containing 5% milk at room temperature for 1 hr and then probed overnight with the following antibodies: anti-GLUT-1 (1:10 000, Abcam, Toronto, On, Canada), anti-GLUT-2 (1:250, Santa Cruz Biotechnology Inc, Mississauga, On, Canada), anti-HIF-1α (1:1000, Abcam, Toronto, On, Canada) and anti-GAPDH (1:10 000, Cell Signaling, Whitby, On, Canada) in PBST containing 1% milk at 4° C. Membranes were washed and then incubated with HRP-conjugated secondary anti-rabbit IgG (1:5000, BD Pharmingen, San Diego, California, USA) antibody at room temperature in PBST containing 1% milk during 1 hr. After extensive washes in PBST, bound peroxidase was detected with enhanced chemiluminescence blotting substrate (Perkin-Elmer, Woodbridge, On, Canada), according to the manufacturer's instructions.

### Triglyceride assay

Measurement of the TG content from cellular and liver specimens was performed by the Metabolomics Core Facility of CRCHUM. Samples were snap-frozen and kept at −80° C until TG determinations. Briefly, lipids from cell pellets were extracted overnight (4° C) in chloroform:methanol (2:1) (Folch extraction [61]). Organic phases (chloroform) were transferred into new glass tubes and dried under nitrogen (N-Evap). Lipids were resuspended in isopropanol and TG were measured enzymatically with the GPO-Trinder kit. Triolein, dissolved in chloroform-methanol and processed similarly to samples, was used as a standard. To normalize the TG content quantification, total protein contents of all samples were measured using the Bradford method.

### Survival analysis

Survival analysis was performed using the PROGgene V2 Prognostic Database (http://watson.compbio.iupui.edu/chirayu/proggene/database/?url=proggene) as described [26, 27]. Each analysis used “liver cancer” as cancer type and “death” as the outcome. The gene expression data was extracted from *The Cancer Genome Atlas* (TCGA) database. The data were not adjusted for clinical status. The survival status was analyzed for expression levels of genes involved in fatty acid biosynthesis, glycolysis and hypoxia-induced response. To analyze the prognostic value of these genes, the Kaplan–Meier method was used to estimate survival curves and the log-rank test was used to compare survival curves of high and low gene expression groups. All the genes studied are listed in Supplementary Table 2.

### Statistical analysis

All data represent the values of at least three independent experiments. Data are expressed as means ± standard error (SEM) and were analyzed with GraphPad Prism7 software. Differences between groups were analyzed using the analysis of variance (ANOVA) test, student *t*-test and Tukey post-test for multiple comparisons. A *P* value below 0.05 was considered significant (^*^=P < 0.05, ^**^= P < 0.01, ^***^= P < 0.001). All statistical tests were two-sided.

## SUPPLEMENTARY MATERIALS FIGURES AND TABLES



## Abbreviations

HCC: Hepatocellular Carcinoma
TG: Triglyceride
PET: Positron Emission Tomography
2-NBDG: 2-[N-(7-nitrobenz-2-oxa-1,3-diaxol-4-yl) amino]-2- deoxyglucose
DHAP: Dihydroxyacetone Phosphate
ECAR: Extracellular Acidification Rate
GLUT-1/2: Glucose Transporter-1/2
AFP: Alfa-fetoprotein
Epcam: Epithelial Cell Adhesion Molecule
Hk II: Hexokinase II
Pfkl: Phosphofructokinase liver
Pdh: Pyruvate dehydrogenase
Pdk1: Pyruvate dehydrogenase kinase 1
Pgc-1α: Peroxisome proliferator-activated receptor gamma coactivator 1-alpha
HIF-1α: Hypoxia Inducible Factor-1α
AMP: Adenosine Monophosphate
ADP: Adenosine Diphosphate
ATP: Adenosine Triphosphate
GMP: Guanosine Monophosphate
GDP: Guanosine Diphosphate
GTP: Guanosine Triphosphate
NAD/H: Nicotinamide Adenine Dinucleotide
NADP/H: Nicotinamide Adenine Dinucleotide Phosphate
GSH: Glutathione
GSSG: Glutathione disulfide
Acly: ATP citrate lyase
Acc: Acetyl-coA carboxylase
Fasn: Fatty acid synthase
TCGA: The Human Cancer Genome Atlas
KM: Kaplan Meier
ROS: Reactive Oxygen Species
