# Supplementary material for: Metabolite profiling identifies a signature of tumorigenicity in hepatocellular carcinoma
Source: Oncotarget. 2018 Jun 1;9(42):26868–83. doi: 10.18632/oncotarget.25525 (PMC6003570; doi:10.18632/oncotarget.25525)
Supplement: Supplementary file 1 [file oncotarget-09-26868-s001.pdf]

Metabolite profiling identifies a signature of tumorigenicity in hepatocellular carcinoma

SUPPLEMENTARY MATERIALS

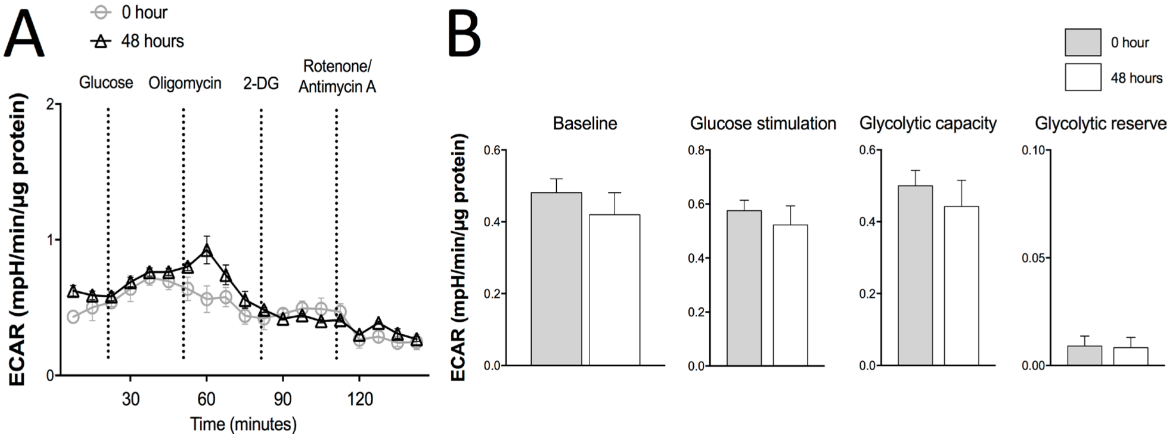

Supplementary Figure 1: Glycolytic profile of primary hepatocytes *in vitro*. (**A–B**) Extracellular acidification rate (ECAR) measurements using the Seahorse XF24 Extracellular Flux analyzer 2 hrs after attachment in freshly isolated (0 hr after attachment) and seeded hepatocytes (48 hrs after attachment) in 25 mM glucose DMEM. Glycolytic capacity and glycolytic reserve were calculated based on the increase in ECAR after injection of oligomycin. Values are ± SEM of at least 3 independent experiments.

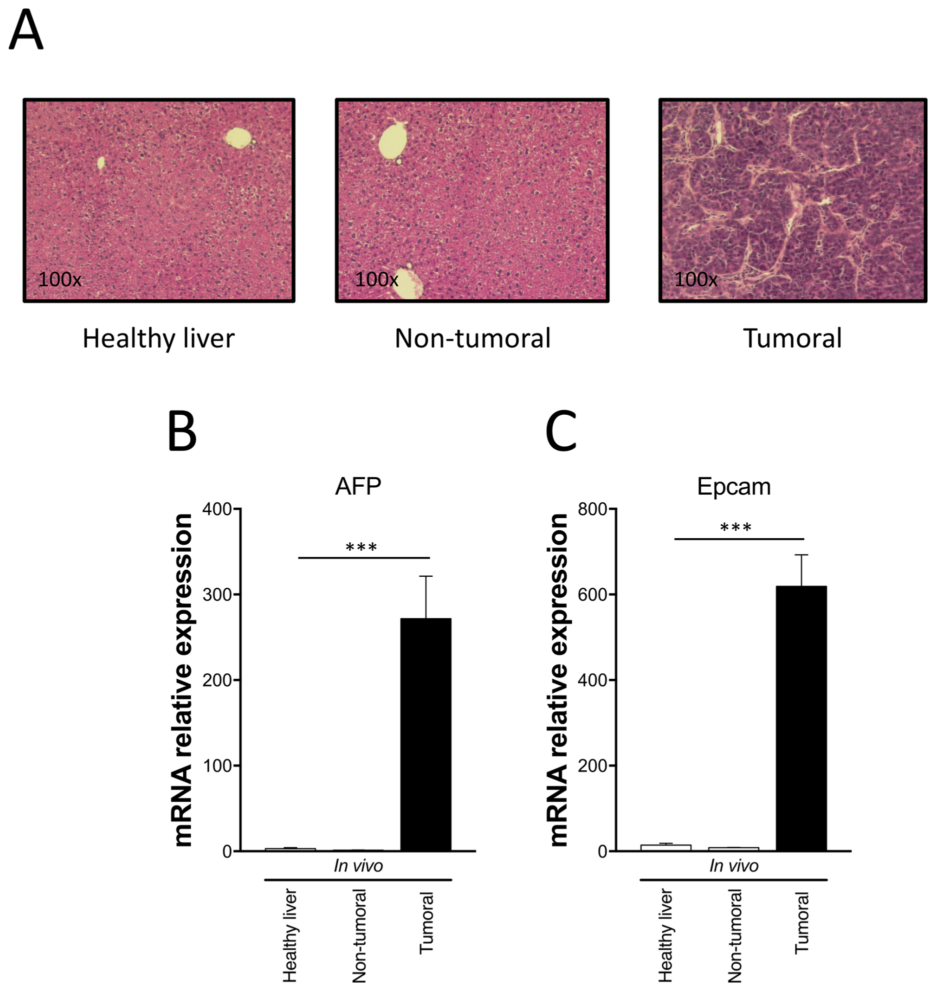

Supplementary Figure 2: Characterization of Dt81Hepa1-6-derived tumoral and non-tumoral liver specimens. (**A**) Representative microphotographs at 100× magnification of HPS-stained liver slices obtained from healthy liver, non-tumoral and tumoral liver specimens. (**B**–**C**) mRNA relative expression of *Alfa-fetoprotein (AFP)* and *Epithelial cell adhesion molecule (Epcam)* in healthy liver, non-tumoral and tumoral liver specimens. Values are ± SEM of at least 3 independent experiments. ($^{***}P < 0.001$).

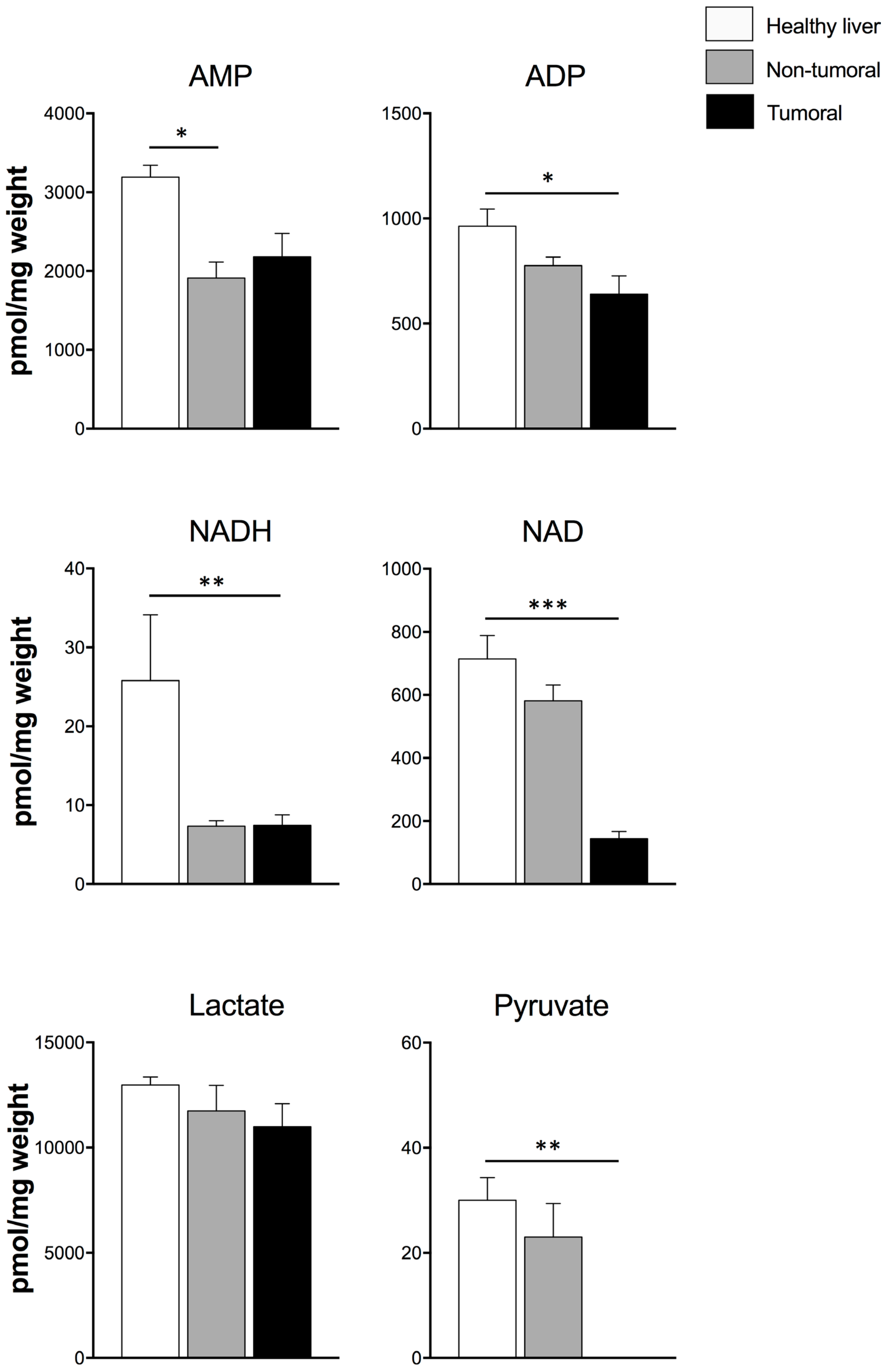

Supplementary Figure 3: Tumorigenic profile of Dt81Hepa1-6-derived tumors. Evaluation of total intracellular AMP, ADP, NADH, NAD, Lactate and Pyruvate in healthy liver, non-tumoral and tumoral liver specimens. Values are ± SEM of at least 3 independent experiments. ($^{*}P < 0.05$, $^{**}P < 0.01$, $^{***}P < 0.001$).

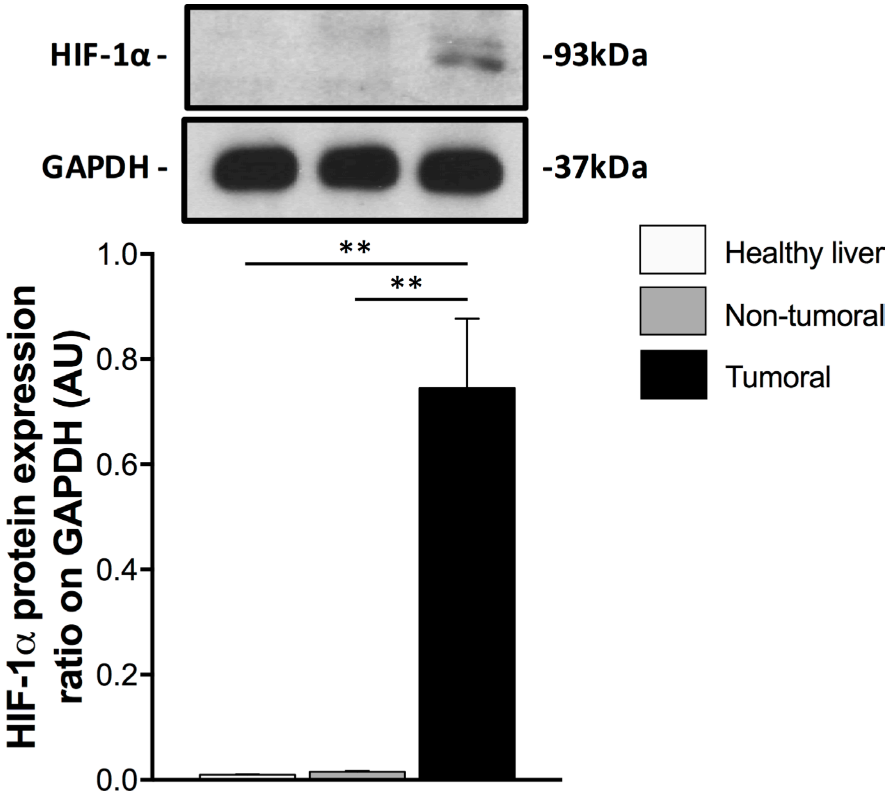

Supplementary Figure 4: Quantification of HIF-1α *in vivo*. HIF-1α protein levels in healthy liver, non-tumoral and tumoral liver specimens. Values are ± SEM of at least 3 independent experiments. (**$P < 0.01$).

Supplementary Table 1: Primer sequences for real-time PCR

Gene	Forward primers	Reverse primers
**Tumor and glycolysis-related molecules**		
Hk II	TGATCGCCTGCTTATTCACGG	AACCGCCTAGAAATCTCCAGA
Pfkl	AGCTCAGAACTACGCACACT	AGTGGTAGTGATGGCGTCAA
Pdh	GGGACGTCTGTTGAGAGAGC	TGTGTCCATGGTAGCGGTAA
Pdk1	CTATGAAAGGCCCCCGTTTT	TAAGTGGACCTCGCAGATGG
Pgc-1α	ATACCGCAAAGAGCACGAGAAG	CTCAAGAGCAGCGAAAGCGTCACAG
HIF-1α	CCAGTTCCTCATCCTTCCCC	ACCGGCATCCAGAAGTTTTC
Cyclin D1	TCCCTGGCTTGCTCAGTGCCTA	TGCCACACGCCATGAGACCA
Afp	TCCTCCTGCTACATTTCGCT	AGGCTTTGCTTGTGCTGAAT
Epcam	AAAGCCAAGCAGTGCAACGGCA	TGTGAACGCCTCTTGAAGCGCA
**Fatty acid metabolism-related molecules**		
Acly	TGGATGCCACAGCTGACTAC	GGTTCAGCAAGGTCAGCTTC
Acc	GAGAGGGGTCAAGTCCTTCC	CTGCTGCCGTCATAAGACAA
Fasn	AAGTTGCCCGAGTCAGAGAA	CGTCGAACTTGGAGAGATCC
**Reference genes**		
HPRT1	GCTTGCTGGTGAAAAGGACCTCTCGAAG	CCCTGAAGTACTCATTATAGTCAAGGGCAT
Ppia	CGCGTCTCCTTCGAGCTGTTTG	TGTAAAGTCACCACCCTGGCACAT
H2afz	ACAGCGCAGCCATCCTGGAGTA	TTCCCGATCAGCGATTTGTGGA

Supplementary Table 2: List of genes used for survival analysis

**Fatty acid biosynthesis pathway**			
Ptges3	*Prostaglandin E Synthase 3*	*Mif*	*Macrophage Migration Inhibitory Factor*
Fads1	*Fatty Acid Desaturase 1*	*Oxsm*	*3-Oxoacyl-ACP Synthase, Mitochondrial*
Ptgs1	*Prostaglandin-Endoperoxide Synthase 1*	*Ptgds*	*Prostaglandin D2 Synthase*
Mcat	*Malonyl-CoA-Acyl Carrier Protein Transacylase*	*Lta4h*	*Leukotriene A4 Hydrolase*
Fads2	*Fatty Acid Desaturase 2*	*Hpgd*	*15-Hydroxyprostaglandin Dehydrogenase*
Cd74	*CD74 Molecule*	*Degs1*	*Delta 4-Desaturase, Sphingolipid 1*
Brca1	*BRCA1, DNA Repair Associated*		
**Glycolysis**			
Eno1	*Enolase 1*	*Pfkm*	*Phosphofructokinase, Muscle*
Eno2	*Enolase 2*	*Pfkp*	*Phosphofructokinase, Platelet*
Eno3	*Enolase 3*	*Pgam1*	*Phosphoglycerate Mutase 1*
Aldoa	*Aldolase, Fructose-Bisphosphate A*	*Pgam2*	*Phosphoglycerate Mutase 2*
Aldob	*Aldolase, Fructose-Bisphosphate B*	*Pgk1*	*Phosphoglycerate Kinase 1*
Aldoc	*Aldolase, Fructose-Bisphosphate C*	*Pklr*	*Pyruvate Kinase, Liver And RBC*
Gapdh	*Glyceraldehyde-3-Phosphate Dehydrogenase*	*Pkm2*	*Pyruvate Kinase, Muscle 2*
Gapdhs	*Glyceraldehyde-3-Phosphate Dehydrogenase, spermatogenic*	*Ppp2ca*	*Protein Phosphatase 2 Catalytic Subunit Alpha*
Gpi	*Glucose-6-Phosphate Isomerase*	*Ppp2cb*	*Protein Phosphatase 2 Catalytic Subunit Beta*
Pfkfb1	*6-Phosphofructo-2-Kinase/Fructose-2,6-Biphosphatase 1*	*Ppp2r1a*	*Protein Phosphatase 2 Scaffold Subunit Aalpha*
Pfkfb2	*6-Phosphofructo-2-Kinase/Fructose-2,6-Biphosphatase 2*	*Ppp2r1b*	*Protein Phosphatase 2 Scaffold Subunit Abeta*
Pfkfb3	*6-Phosphofructo-2-Kinase/Fructose-2,6-Biphosphatase 3*	*Ppp2r5d*	*Protein Phosphatase 2 Regulatory Subunit B'Delta*
Pfkfb4	*6-Phosphofructo-2-Kinase/Fructose-2,6-Biphosphatase 4*	*Tpi1*	*Triosephosphate Isomerase 1*
Pfkl	*Phosphofructokinase, Liver Type*		
**Hypoxia-induced response**			
Cldn3	*Claudin 3*	*Cd24*	*CD24 Molecule*
Pdia2	*Protein Disulfide Isomerase Family A Member 2*	*Mt3*	*Metallothionein 3*
Arnt2	*Aryl Hydrocarbon Receptor Nuclear Translocator 2*	*Epas1*	*Endothelial PAS Domain Protein 1*
Pml	*Promyelocytic Leukemia*	*Nf1*	*Neurofibromin 1*
Egln2	*Egl-9 Family Hypoxia Inducible Factor 2*	*Crebbp*	*CREB Binding Protein*
Bnip3	*BCL2 Interacting Protein 3*	*Smad4*	*SMAD Family Member 4*
Egln1	*Egl-9 Family Hypoxia Inducible Factor 2*	*Smad3*	*SMAD Family Member 3*
Tgfb2	*Transforming Growth Factor Beta 2*	*Hsp90b1*	*Heat Shock Protein 90 Beta Family Member 1*
Alas2	*5'-Aminolevulinate Synthase 2*	*Ep300*	*E1A Binding Protein P300*
Plod1	*Procollagen-Lysine,2-Oxoglutarate 5-Dioxygenase 1*	*Hif1a*	*Hypoxia Inducible Factor 1 Alpha Subunit*
Plod2	*Procollagen-Lysine,2-Oxoglutarate 5-Dioxygenase 2*	*Vegfa*	*Vascular Endothelial Growth Factor A*
Cxcr4	*C-X-C Motif Chemokine Receptor 4*	*Narfl*	*Nuclear Prelamin A Recognition Factor Like*
Ang	*Angiogenin*	*Chrnb2*	*Cholinergic Receptor Nicotinic Beta 2 Subunit*
Chrna4	*Cholinergic Receptor Nicotinic Alpha 4 Subunit*		
